# Autophagy and mitophagy biomarkers are reduced in sera of patients with Alzheimer’s disease and mild cognitive impairment

**DOI:** 10.1038/s41598-019-56614-5

**Published:** 2019-12-27

**Authors:** Massimiliano Castellazzi, Simone Patergnani, Mariapina Donadio, Carlotta Giorgi, Massimo Bonora, Cristina Bosi, Gloria Brombo, Maura Pugliatti, Davide Seripa, Giovanni Zuliani, Paolo Pinton

**Affiliations:** 1 Department of Biomedical and Specialist Surgical Sciences, section of Neurological, Psychiatric and Psychological sciences, University of Ferrara, Ferrara, Italy; 2 Interdepartmental Research Center for the Study of Multiple Sclerosis and Inflammatory and Degenerative Diseases of the Nervous System, University of Ferrara, Ferrara, Italy; 3 Department of Morphology, Surgery and Experimental Medicine, Section of Pathology, Oncology and Experimental Biology, Laboratory for Technologies of Advanced Therapies (LTTA), University of Ferrara, Ferrara, Italy; 4 Maria Cecilia Hospital, GVM Care & Research, Cotignola, Ravenna Italy; 5 Department of Morphology, Surgery and Experimental Medicine, Section of Anatomic Pathology, University of Ferrara, Ferrara, Italy; 6 Department of Morphology, Surgery and Experimental Medicine, Section of Internal Medicine, University of Ferrara, Ferrara, Italy; 7 Gerontology and Geriatric Research Laboratory, IRCCS Casa Sollievo della Sofferenza, San Giovanni Rotondo, Foggia, Italy

**Keywords:** Cognitive ageing, Geriatrics

## Abstract

Dementia is a neurocognitive disorder characterized by a progressive memory loss and impairment in cognitive and functional abilities. Autophagy and mitophagy are two important cellular processes by which the damaged intracellular components are degraded by lysosomes. To investigate the contribution of autophagy and mitophagy in degenerative diseases, we investigated the serum levels of specific autophagic markers (ATG5 protein) and mitophagic markers (Parkin protein) in a population of older patients by enzyme-linked immunosorbent assay. Two hundred elderly (≥65 years) outpatients were included in the study: 40 (20 F and 20 M) with mild-moderate late onset Alzheimer’s disease (AD); 40 (20 F and 20 M) affected by vascular dementia (VAD); 40 with mild cognitive impairment (MCI); 40 (20 F and 20 M) with “mixed” dementia (MD); 40 subjects without signs of cognitive impairment were included as sex-matched controls. Our data indicated that, in serum samples, ATG5 and Parkin were both elevated in controls, and that VAD compared with AD, MCI and MD (all p < 0.01). Patients affected by AD, MD, and MCI showed significantly reduced circulating levels of both ATG5 and Parkin compared to healthy controls and VAD individuals, reflecting a significant down-regulation of autophagy and mitophagy pathways in these groups of patients. The measurement of serum levels of ATG5 and Parkin may represent an easily accessible diagnostic tool for the early monitoring of patients with cognitive decline.

## Introduction

Dementia is a major neurocognitive disorder characterized by a progressive memory loss and impairment in cognitive and functional abilities^1^; in dementia, the parts of the brain designated to memory, learning, and decision making are damaged or diseased. Dementia comes in different forms, Alzheimer’s and vascular being the most common types, and is often preceded by a mild cognitive impairment (MCI) stage, a condition described as a transition phase between normal cognitive ageing and dementia^1^. However, diagnosis and differentiation of dementia types requires a careful examination, especially in the elderly, since clinical and pathological findings often overlap.

Autophagy is an important cellular process by which the damaged intracellular components are sequestered, degraded and digested by lysosomes^2^. Regulation and maintenance of this process is crucial for both cellular homeostasis through the elimination and recycling of the constituents of the cell itself, and the maintenance of cellular integrity, in order to prevent the accumulation of misfolded proteins or damaged/malfunctioning organelles^3^. Autophagy has been extensively studied in last decade and a series of specific genes related to this process (ATG genes) has been unveiled^4^. Autophagy also exists in different selective forms. The most studied and relevant for human diseases is mitophagy, which is responsible for the removal of aberrant and aged mitochondria^3^. A specific molecular axis has been found to regulate this selective form of autophagy. Accordingly, two genes associated with Parkinson’s disease, mitochondrial kinase PTEN-induced kinase 1 (PINK1) and ubiquitin ligase Parkin, recognize damaged mitochondria and label them for autophagic degradation^5^. In particular, Parkin is an ubiquitin ligase that is recruited from the cytosol to depolarize mitochondria to mediate its selective removal^6^.

Autophagy and mitophagy are implicated in various neurodegenerative diseases, such as Alzheimer’s disease, Parkinson’s disease (PD) and amyotrophic lateral sclerosis (ALS), where they exert protective roles by removing abnormal aggregated proteins^7^. Accordingly, an alteration in autophagic and mitophagic pathways has been found in post-mortem analysis of human brain tissues and in experimental animal models of neurodegenerative diseases^8^.

The contribution of autophagy and mitophagy in degenerative pathologies of the central nervous system (CNS) is quite evident. Despite this, at present, the possible contribution of autophagy and mitophagy to dementia remains to be elucidated.

To investigate this topic, we studied the serum levels of specific autophagic markers, (ATG5 protein) and the mitophagic marker (Parkin protein), in a sample of both older patients affected by various forms of dementia and controls.

## Materials and Methods

### Study design and sample handling

Two hundred elderly (≥65 years) outpatients admitted from 2016 to 2018 to the Memory Clinics of *Casa Sollievo della Sofferenza*, San Giovanni Rotondo or to the Department of Internal Medicine, S. Anna University Hospital, Ferrara (Italy) were included in the study:

- forty patients, 20 female and 20 male, with mild-moderate late onset Alzheimer’s disease (AD), according to the NINCDS-ADRDA criteria^9^: Mini Mental State Examination (MMSE) range: 18–23; Clinical Dementia Rating (CDR) score range: 1–2;
- forty patients, 20 female and 20 male, affected by vascular dementia (VAD) defined by the NINDS-AIREN criteria^10^: MMSE range: 18–24; CDR: 1–2;
- forty patients, 20 female and 20 male, with mild cognitive impairment (MCI), defined as presence of short/long-term memory impairment, with or without impairment in other single or multiple cognitive domains, in individuals who did not meet the standardized criteria for dementia^11^: MMSE range: 23–26. Most of these individuals were affected by amnestic multidomain MCI;
- forty patients with “mixed” dementia (MD), 20 female and 20 male. In these patients, a definite diagnosis of AD or VD was not possible since both the clinical characteristics of late-onset Alzheimer disease (LOAD) and VAD were present (CT/MRI demonstrated significant cerebrovascular disease, but the evolution of symptoms was slow and progressive): MMSE range: 18–23; CDR: 1–2;
- forty subjects without signs or symptoms of cognitive impairment were included as sex-matched controls.

All the procedures performed in our studies involving human participants were in accordance with the ethical standards of the institutional and/or national research committee and with the 1964 Helsinki declaration and its later amendments or comparable ethical standards. All participants (and their caregivers if demented) were informed about the research project and signed an informed consent. The study was approved by the Local Ethic Committee of “Casa Sollievo della Sofferenza”, San Giovanni Rotondo (protocol n. 3877/DS) and the Local Ethic Committee of “Azienda Arcispedale S. Anna”, Ferrara (protocol n. 170579). The entire research study was performed in accordance with the relevant guidelines and regulations. In detail:

- Subjects affected by severe congestive heart failure (NYHA class III-IV), severe liver or kidney disease, severe chronic obstructive pulmonary disease (COPD), and cancer were excluded.
- There was no evidences of acute illnesses at the time of enrollment.
- No subject was taking nonsteroidal anti-inflammatory drugs, antibiotics, or steroids. Personal data and medical history were collected by trained personnel.
- General and neuropsychological examination was carried out as previously described^12^.
- Clinical chemistry analyses were routinely performed to exclude causes of secondary cognitive impairment.
- Trained geriatricians made the diagnosis of dementia as previously described^13^.
- Venous blood was withdrawn from all subjects as part of the diagnostic work-up.
- All samples were taken, stored and analyzed in the same conditions. To obtain serum samples, blood was subjected to centrifugation at 3,000 rpm at 20 °C for 15 minutes. Supernatants were then collected, under sterile conditions, in aliquots of 500 μl, coded, frozen and stored at −80 °C until assay.

### Serum levels of ATG5 and parkin determination

Serum concentrations of ATG5 and Parkin were determined by using commercially available enzyme-linked immunosorbent assay kits (My Biosource, San Diego, California, USA; MS7209535 for ATG5 and MBS732278 for Parkin) following the manufacturer’s instructions as previously published^14,15^.

### Data analysis

Statistical analysis was performed with GraphPad Prism®. Normal distribution was tested by means of the Kolmogorov-Smirnov test. As different variables did not match the normality assumption, multiple comparisons were performed through the nonparametric Kruskal-Wallis test with Dunn’s test correction. A value of p < 0.05 was accepted as statistically significant.

### Ethics approval and consent to participate

The study was approved by the local Committee for Medical Ethics in Research, and written consent to study participation was obtained from all subjects.

## Results

The study was conducted on 200 elderly individuals: 160 patients with different kinds of cognitive impairment (40 AD, 40 MCI, 40 VD, 40 MD), and 40 control subjects. The main clinical-demographic features of the study population are reported in Table 1. The gender ratio was set at 1 in all patient subgroups.

**Table 1:** Demographic and clinical main features of study population.

	Controls		VAD		AD		MD		MCI	
	F (n = 20)	M (n = 20)	F (n = 20)	M (n = 20)	F (n = 20)	M (n = 20)	F (n = 20)	M (n = 20)	F (n = 20)	M (n = 20)
Age, years: mean ± SD	75 ± 8	75 ± 7	80 ± 6	77 ± 7	79.5 ± 5	79 ± 6	80 ± 5	79 ± 4	77.5 ± 5	77 ± 6
Education, years: mean ± SD	8 ± 4	10 ± 5	4 ± 3	8 ± 4	5 ± 3	7 ± 4	6 ± 3	7 ± 4	6 ± 3	7.5 ± 4
**Comorbidities:**										
Hypertension, yes: N (%)	14 (70%)	12 (60%)	16 (80%)	14 (70%)	14 (70%)	11 (55%)	13 (65%)	14 (70%)	13 (65%)	13 (65%)
Diabetes, yes: N (%)	2 (10%)	4 (20%)	5 (25%)	5 (25%)	3 (15%)	3 (15%)	3 (15%)	4 (20%)	2 (10%)	5 (25%)
CHD, yes: N (%)	1 (5%)	4 (20%)	4 (20%)	5 (25%)	2 (10%)	4 (20%)	2 (10%)	7 (35%)	2 (10%)	4 (20%)
Stroke, yes: N (%)	0 (0%)	1 (5%)	0 (0%)	5 (25%)	0 (0%)	0 (0%)	0 (0%)	2 (10%)	0 (0%)	2 (10%)
COPD, yes: N (%)	1 (5%)	2 (10%)	1 (5%)	2 (10%)	1 (5%)	3 (15%)	1 (5%)	3 (15%)	2 (10%)	2 (10%)

AD, Alzheimer’s disease; CHD, coronary heart disease; COPD, chronic obstructive pulmonary disease, F, female; M, male; MCI, mild cognitive impairment; MD, “mixed” dementia; SD, standard deviation; VAD, vascular dementia.

### Serum levels of ATG5

**Figure 1:**
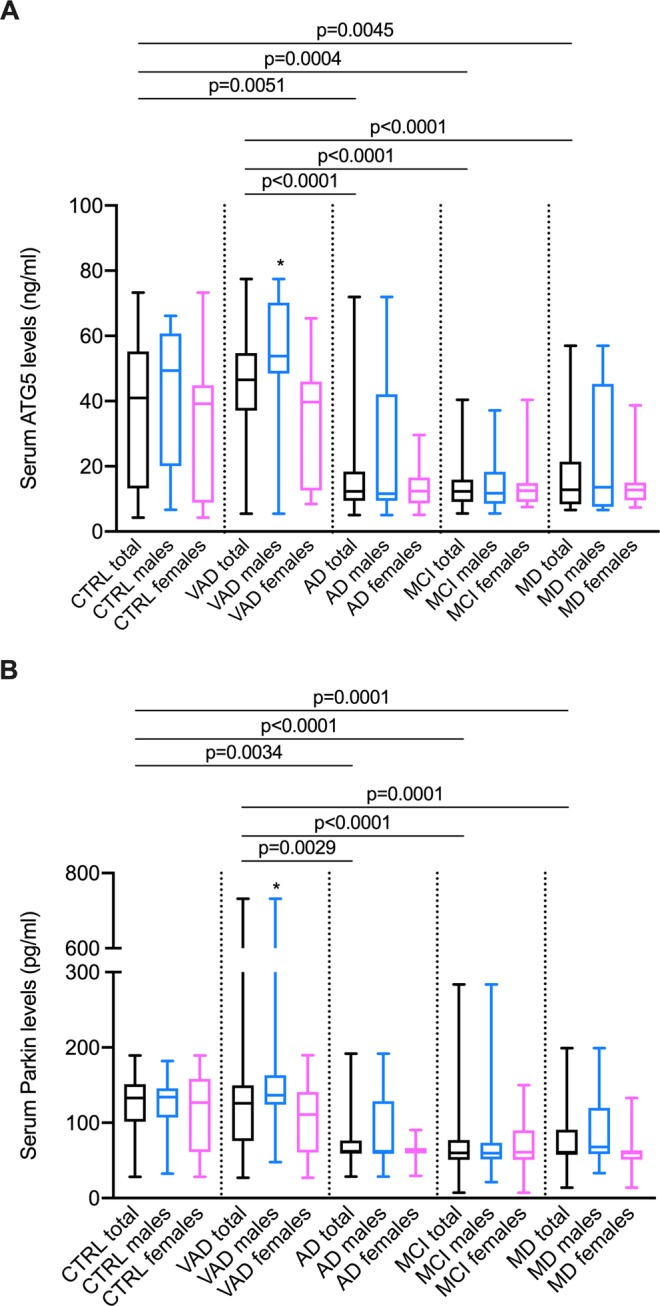
Serum levels of ATG5 and Parkin in sera of patients affected by Alzheimer’s disease (AD), vascular dementia (VAD), mild cognitive impairment (MCI), “mixed” dementia (MD) and without signs of cognitive impairment as sex-matched controls. Panel A: ATG5 levels were different among groups (Kruskall-Wallis; p < 0.0001), in particular in post hoc analysis (Dunn’s post hoc test) median ATG5 values were more elevated in Controls and VAD than in AD (p = 0.0051 and p < 0.0001), MCI (p = 0.0004 and p < 0.0001) and MD (p = 0.0045 and p < 0.0001). Panel B: Parkin levels were different among groups (Kruskall-Wallis; p < 0.0001), post hoc analysis (Dunn’s post hoc test) showed higher median values in Controls and VAD respect to AD (p = 0.0034 and p = 0.0029), MCI (both p < 0.0001) and MD (both p = 0.0001). *After stratification by sex, ATG5 and Parkin value resulted augmented in males compared to females in VAD subgroup (Mann-Whitney; p < 0.0001 and p = 0.0245, respectively). The boundaries of the boxes represent the 25th–75th quartile. The line within the box indicates the median. The vertical lines above and below the box correspond to the highest and lowest values.

Serum levels of ATG5 were statistically different among the various groups of patients (Kruskal-Wallis test: p < 0.0001). Post hoc analysis revealed that median serum concentrations of ATG5 were elevated in controls (45.95 ng/ml) and VAD (46.57 ng/ml) compared with AD (12.35 ng/ml), MCI (12.32 ng/ml) and MD (12.84 ng/ml) (all p < 0.01) (Fig. 1, Panel A). After stratification by sex, median serum levels of ATG5 were found to be higher in males (53.80 ng/ml) than in females (39.72 ng/ml) in the VAD subgroup. No further statistically significant sex-related differences were found.

### Serum levels of parkin

Serum concentrations of Parkin were statistically different among the various groups of patients (Kruskal-Wallis test: p < 0.0001). Post hoc analysis revealed that median serum levels of Parkin were more elevated in controls (132.8 pg/ml) and VAD (125.9 pg/ml) as compared to AD (62.13 pg/ml), MCI (59.89 pg/ml), and MD (60.80 pg/ml) (all p < 0.01) (Fig. 1, Panel B). After stratification by sex, median serum concentrations of Parkin resulted higher in males (136.7 pg/ml) than in females (111.0 pg/ml) in the VAD subgroup. No further statistically significant sex-related differences were found.

## Discussion

In this study, for the first time, the presence of specific autophagic (ATG5 protein) and mitophagic (Parkin protein) markers were investigated in the serum of both patients affected by different types of cognitive decline and in sex-matched healthy individuals. Patients affected by AD, MD, and MCI showed significantly reduced circulating levels of both ATG5 and Parkin compared to healthy controls and VAD individuals. These findings were also confirmed by measuring the amounts of the other autophagic related gene Beclin-1 (Fig. S1), a factor known to play a central role in autophagosome formation, as well as in the initiation of mitophagy following interaction with Parkin^16,17^. Overall, our results show that these groups of patients are characterized by a deep down-regulation of autophagy and mitophagy. In addition, we have demonstrated a sex-related difference in the expression of auto/mitophagic markers in patients suffering from VAD with a significant reduction in females compared to males.

From the anatomopathological point of view, the brain of AD patients is characterized by the presence of extracellular amyloid plaques (Aβ) and intraneuronal neurofibrillary tangles consisting of aggregated tau protein^18^. Differently, MCI individuals not only possess amyloid plaques and neurofibrillary tau tangles, but also cerebral vascular disease such as arteriosclerosis and cerebral amyloid angiopathy (CAA) that, however, represent an independent risk factor for dementia. In this perspective, the facilitation of Aβ clearance could be a potential treatment for AD and MCI^19^. Although AD is classified as a neurodegenerative disease, studies have shown strong links between AD and cerebrovascular disease (CVD). Vascular risk factors and CVD can indeed accelerate the production and deposition of Aβ contributing to the onset and progression of AD^20^.

In the last decade, it has been suggested that autophagy makes an important contribution to the arrangement of different CVD, participating in the mechanism of both Aβ and tau aggregate formation^21,22^. For example, it has been found that Aβ peptide and the amyloid precursor protein levels can be reduced by autophagy throughout ATG5 activity^23^. Moreover, it has been observed that when a dysfunction of the autophagic process occurs, the correct clearance of these aggregates fails. In confirmation of this, altered functions of specific autophagic genes have been found in 1) cellular models of AD, 2) transgenic mouse models that mimic a range of Alzheimer’s disease–related pathologies and 3) in human brain samples obtained from AD-affected patients^24–26^. Finally, mitochondria cover a crucial role in regulating Aβ and tau aggregates. Indeed, mitochondrial dysfunctions and associated oxidative stress, synergically with age-related factors (such as an inactive lifestyle, or excessive caloric intake), increase the inflammatory process and promote amyloidogenic APP processing^27^. Accordingly, studies in living AD patients and postmortem brain tissue of AD-affected individuals, have provided evidence that neurons suffer from impaired mitochondrial function. Overall, maintaining a functional mitochondrial population seems to be fundamental during AD pathogenesis. Hence, compromised mitophagy may result in the accumulation of dysfunctional mitochondria in AD. Recently, several studies have identified defective mitophagy and mitochondrial dysfunctions in postmortem tissue samples from AD subjects and in AD patients’ induced pluripotent stem cell (iPSC)-derived cultured neurons^28,29^. Conversely, restoration of neuronal mitophagy was found sufficient to ameliorate the cognitive decline in an AD mouse model by preventing synaptic failure. In particular, it was proven that, improving the activity of a master-regulator of mitophagy, Parkin, the autophagic clearance of Aβ and Tau into the lysosome is favored^30^. Despite these findings, very poor correlations between autophagic/mitophagic pathways and dementia have been reported.

Our results confirm that a deficit of autophagy and mitophagy are associated with those forms of dementia which are associated with accumulation of Aβ and tau proteins (i.e. AD and MD). Moreover, our results suggest that such deficit is already present in subjects with mild cognitive impairment. In fact, MCI individuals, characterized by an intermediate condition between normal cognitive ageing and dementia, displayed similar levels of both ATG5 and Parkin compared with AD and MD. Overall, these data suggest that a deregulation of auto/mitophagic processes may be an important element in the early stage of AD, which is maintained during progression of the disease.

Our data also indicate that this catabolic deficit of auto/mitophagy is not present in patients with VAD. Vascular dementia is widely regarded as the second most common type of dementia^31^. VAD can culminate with global or focal effects on vascular disease, and is characterized by cognitive impairment, often incorporating behavioral symptoms and locomotor abnormalities. Within the VAD spectrum, the most common vascular contribution to dementia is represented by cerebral small vessel disease (cSVD) syndrome^32^, whose frequency is increasing nowadays due to longer survival of the elderly population^33^. Our results seem to be in contrast with a recent publication where Cho and colleagues described an increasing expression of ATG5 in plasma samples of dementia-affected individuals^34^. Nevertheless, in this study the authors did not group the patients according to the dementia sub-type as we did. In fact, our results have highlighted for the first time that autophagy plays different roles in the sub-types of dementia, with a significant reduction in AD, MCI and MD compared to VD.

On the sidelines of the main results that emerged from our study, we report for the first time a sex-related different expression of auto/mitophagic biomarkers in patients affected by VAD. Females and males have different incidence and symptom presentation of both dementia and vascular diseases^35,36^. Moreover, sex differences in autophagic mechanisms have been proposed as a contributory factor to female vulnerability in AD^37^. On the one hand, our results suggest a gender role in the predisposition to deficient auto/mitophagy during VAD, on the other, they underline the importance of the study design in avoiding imbalances in the readouts caused by gender and not in the expression of the molecules themselves.

Together with the pathogenic role of autophagy and mitophagy in the onset and progression of cognitive disorders, our results also highlight the role of ATG5 and Parkin as potential biological markers of cognitive impairment. Indeed, while Aβ and tau are disease markers at the level of the central nervous system, as they accumulate at the site where neurodegeneration is taking place, ATG5 and Parkin also seem to be reduced at a systemic level, making it easier to measure them through a simple blood sample. This opens up the future possibility of using these molecules as early markers of dementia in order to distinguish between AD-type dementias and VAD.

The main limitation of our study is the lack of a comparison between the levels of ATG5 and Parkin with other molecular or neuroimaging biomarkers. However, clinical classification, sample size, and equal gender distribution in the various groups taken together should give consistency to our results.

## Conclusions

Our data highlight an important reduction in the levels of markers of auto- and mitophagy in patients suffering from neurodegenerative cognitive impairment such as AD, MD, and MCI compared to healthy controls and patients with VAD. Furthermore, the measurement of serum levels of ATG5 and Parkin, as biomarkers of autophagy and mitophagy, respectively, may represent an easily accessible diagnostic tool for the early monitoring of patients with cognitive decline.

## Supplementary information

Supplementary information

## Data Availability

The materials used in the current study are available from the corresponding author on reasonable request.
