# Supplementary material for: Autophagy and mitophagy biomarkers are reduced in sera of patients with Alzheimer’s disease and mild cognitive impairment
Source: Sci Rep. 2019 Dec 27;9:20009. doi: 10.1038/s41598-019-56614-5 (PMC6934625; doi:10.1038/s41598-019-56614-5)
Supplement: Supplementary file 1 — Supplementary information [file 41598_2019_56614_MOESM1_ESM.pdf]

SUPPLEMENTARY INFORMATIONS

TITLE

Autophagy and mitophagy biomarkers are reduced in sera of patients with Alzheimer's disease and mild cognitive impairment

AUTHORS

Massimiliano Castellazzi,[1,2,*] Simone Patergnani,[3,4,*] Mariapina Donadio,[1] Carlotta Giorgi,[3] Massimo Bonora,[3,] Cristina Bosi,[5] Gloria Brombo,[6] Maura Pugliatti,[1,2] Davide Seripa, [7,] Giovanni Zuliani,[6,§] Paolo Pinton,[3,4,§]

[1]Department of Biomedical and Specialist Surgical Sciences, section of Neurological, Psychiatric and Psychological sciences, University of Ferrara, Ferrara, Italy
[2]Interdepartmental Research Center for the Study of Multiple Sclerosis and Inflammatory and Degenerative Diseases of the Nervous System, University of Ferrara, Ferrara, Italy
[3]Department of Morphology, Surgery and Experimental Medicine, Section of Pathology, Oncology and Experimental Biology, Laboratory for Technologies of Advanced Therapies (LTTA), University of Ferrara, Ferrara, Italy
[4]Maria Cecilia Hospital, GVM Care & Research, Cotignola, Ravenna, Italy;
[5]Department of Morphology, Surgery and Experimental Medicine, Section of Anatomic Pathology, University of Ferrara, Ferrara, Italy
[6]Department of Morphology, Surgery and Experimental Medicine, Section of Internal Medicine, University of Ferrara, Ferrara, Italy
[7]Gerontology and Geriatric Research Laboratory, IRCCS Casa Sollievo della Sofferenza, San Giovanni Rotondo, Foggia, Italy

[*]equally contributed

[§]Corresponding author:
Professor Paolo Pinton, Department of Morphology, surgery and experimental Medicine, section of Pathology, Oncology and experimental Biology, Laboratory for technologies of Advanced therapies (LTTA), University of Ferrara, Ferrara, Italy; paolo.pinton@unife.it
Professor Giovanni Zuliani, Department of Morphology, Surgery and Experimental Medicine, Section of Internal Medicine, University of Ferrara, Ferrara, Italy; giovanni.zuliani@unife.it

To confirm the role of auto/mitophagy in our population of patients, another molecule, Beclin-1 [1,2] was measured in 80 subjects: 16 patients (8 females and 8 males) were analyzed for each subgroup.

Materials and methods

Serum levels of Beclin-1 determination

Serum concentrations of Beclin-1 were determined by using commercially available enzyme-linked immunosorbent assay kits (My Biosource MBS732891, San Diego, California, USA) following the manufacturer's instructions.

Results

Serum levels of Beclin-1

As reported in figure 2 (panel A) serum levels of Beclin-1 were statistically different among the groups (Kruskal-Wallis test: $p<0.0001$). In particular, serum median levels of Beclin-1 were more elevated in controls (53.69 ng/ml) than in AD (19.84 ng/ml), MD (30.47 ng/ml) and MCI (28.88 ng/ml) (Dunn's post hoc test: $p=0.0004$, $p=0.0471$ and $p=0.0253$, respectively) and in VAD and VAD (69.93 ng/ml) than in AD, MD and MCI (Dunn's post hoc test: all $p<0.0001$). Moreover, in all patients analyzed as a whole, serum levels of Beclin-1 were positively correlated to Parkin ($r=0.5786$) and to ATG5 ($r=0.5318$) (Spearman: both $p<0.0001$) (Figure 2, Panels B and C).

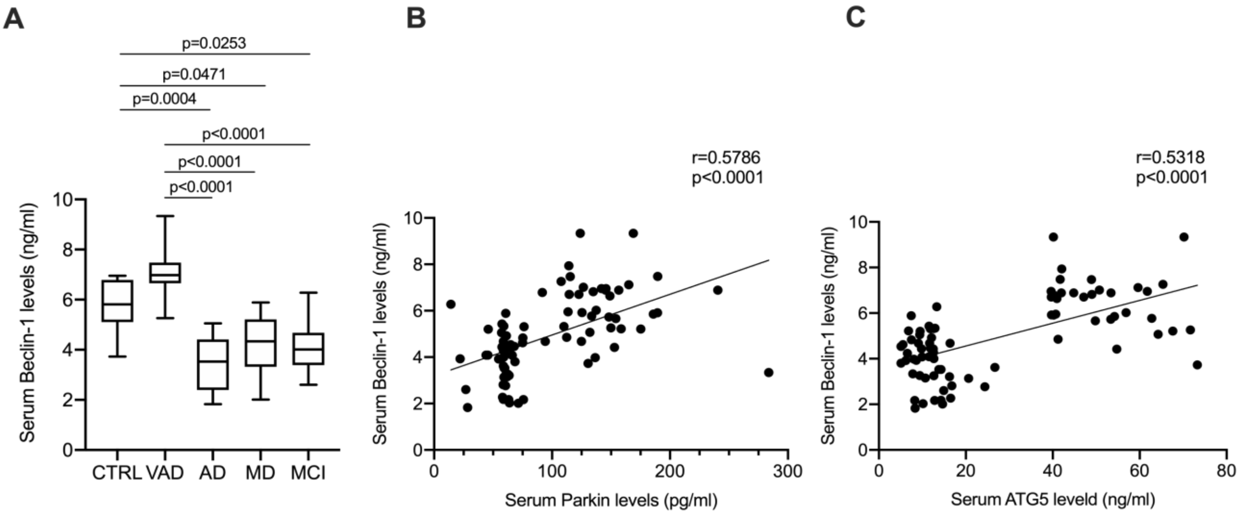

Supplementary Figure 1.

Serum levels of Beclin-1 in sera of patients affected by Alzheimer's disease (AD), vascular dementia (VAD), mild cognitive impairment (MCI), "mixed" dementia (MD) and without signs of cognitive impairment as sex-matched controls. **Panel A**: Beclin-1 levels were different among groups (Kruskall-Wallis; $p<0.0001$), in particular in post hoc analysis (Dunn's post hoc test) median ATG5 values were more elevated in Controls and VAD than in AD ($p=0.0004$ and $p<0.0001$), MCI ($p=0.0253$ and $p<0.0001$) and MD ($p=0.0471$ and $p<0.0001$). **Panel B** and **C**: Beclin-1 levels were positively correlated to Parkin and ATG5 concentrations ($r=0.5786$ and $r=0.5318$, respectively) (Spearman: both $p<0.0001$) in the patient population analyzed as a whole.

References for Supplementary Information

1 Liang, X. H. *et al.* Induction of autophagy and inhibition of tumorigenesis by beclin 1. *Nature* **402**, 672-676, doi:10.1038/45257 (1999).

2 Hill, S. M., Wrobel, L. & Rubinsztein, D. C. Correction to: Post-translational modifications of Beclin 1 provide multiple strategies for autophagy regulation. *Cell death and differentiation* **26**, 2810, doi:10.1038/s41418-019-0346-1 (2019).
